# Video-Assisted Thoracoscopic Surgery with Bullectomy and Partial Pleurectomy versus Chest Tube Drainage for Treatment of Secondary Spontaneous Pneumothorax—A Retrospective Single-Center Analysis

**DOI:** 10.3390/medicina58030354

**Published:** 2022-02-27

**Authors:** Stephen Fung, Marius Kivilis, Andreas Krieg, Anja Schauer, Alexander Rehders, Levent Dizdar, Wolfram-Trudo Knoefel

**Affiliations:** Department of Surgery, University Hospital Duesseldorf and Heinrich-Heine-University Duesseldorf, 40225 Duesseldorf, Germany; stephen.fung@med.uni-duesseldorf.de (S.F.); marius.kivilis@med.uni-duesseldorf.de (M.K.); andreas.krieg@med.uni-duesseldorf.de (A.K.); anjamaria.schauer@med.uni-duesseldorf.de (A.S.); rehders@med.uni-duesseldorf.de (A.R.); levent.dizdar@med.uni-duesseldorf.de (L.D.)

**Keywords:** VATS-bullectomy, partial pleurectomy, chest tube, recurrence, SSP

## Abstract

*Background and objective:* Current guidelines recommend chest tube (CT) drainage as the initial treatment of secondary spontaneous pneumothorax (SSP). Surgery should be considered in cases of persistent air leak or recurrent disease. Video-assisted thoracoscopic surgery (VATS) is nowadays an established surgical treatment for complicated spontaneous pneumothorax. However, reports on VATS-bullectomy with partial pleurectomy (VBPP) for treatment of secondary spontaneous pneumothorax (SSP) are limited. The primary aim of this study was to evaluate and compare the clinical outcomes of patients with secondary pneumothorax treated either by VBPP or CT drainage in our institution. Secondly, we assessed underlying clinical parameters to identify potential risk factors for SSP recurrence. *Materials and Methods:* Eighty-two patients were included in this study. Long-term recurrence rates and potential risk factors for SSP recurrence were analyzed. *Results:* Thirty-six patients (43.9%) underwent VBPP, whereas 46 (56.1%) patients subsequently underwent CT treatment. During a median follow-up period of 76.5 months, VBPP patients experienced a significantly low recurrence rate compared to CT patients (VBPP vs. CT: 16.7% vs. 41.3%; *p* = 0.016). However, VBPP was associated with a higher complication rate and significantly longer length of hospital stay (LOS). Male sex (male vs. female: *p* = 0.021) and CT treatment (VBPP vs. CT: *p* < 0.001) were identified as potential risk factors for SSP recurrence. *Conclusions:* VBPP is a suitable surgical treatment for SSP. However, prolonged LOS and possible complications should be discussed prior to VBPP.

## 1. Introduction

Spontaneous pneumothorax (SP) describes the presence of air without preceding trauma within the pleural space. SP in patients with an underlying pulmonary disease, commonly chronic obstructive pulmonary disease (COPD), is classified as secondary spontaneous pneumothorax (SSP). In most cases, the patients are 45 years of age or more [1]. The incidence of SSP has been reported with approximately 2.0 and 6.3 cases per 100,000 individuals per year in females and males, respectively [2].

In contrast to patients with primary spontaneous pneumothorax (PSP), secondary spontaneous pneumothorax is a potential life-threatening condition due to its cardiopulmonary compromise [3,4]. Therefore, immediate diagnosis and treatment of SSP is mandatory. At the initial presentation of SSP, current guidelines recommend, depending on the patient’s clinical condition, oxygen supplementation, needle-aspiration, and chest tube (CT) drainage [1,5,6]. Despite high rates of treatment failure, CT drainage is still the most commonly used initial treatment [1,5,6]. In most cases, surgery is recommended for persistent air leak after CT drain placement and for patients with recurrent disease. However, due to the underlying clinical disease, patients with SSP are more likely to experience treatment failure and in-hospital mortality even after surgery [7,8].

In previous studies [3,9,10], surgery for SSP (VATS-talc pleurodesis, open thoracotomy, VATS-apical pleurectomy, VATS-pleural abrasion) was reported to be associated with a reduced risk of recurrence. However, data reporting the outcome of SSP after VATS-bullectomy with partial pleurectomy (VBPP) are limited. Moreover, studies that evaluate risk factors for SSP recurrence are lacking.

Therefore, the primary aim of this study was to evaluate and compare the outcomes and long-term recurrence rates of patients following VBPP and CT treatment in our institution. Secondly, we analyzed underlying clinical factors to determine potential predictors for SSP recurrence in our patient cohort.

## 2. Materials and Methods

We retrospectively reviewed the data of 82 patients with secondary spontaneous pneumothorax (SSP), treated either by VATS-bullectomy with partial pleurectomy (VBPP) or by chest tube (CT) only between January 2008 and December 2020 in our institution. Patient demographics, including age, sex, body mass index (BMI), COPD stage, ECOG status, Charlson Comorbidity Index score, treatment modality, etiological cause of SSP, post-treatment complications, length of hospital stay (LOS), and size of the pneumothorax, were retrieved from medical records. The size of the pneumothorax was assessed using the regression formula derived from the method of Collins [11]. According to the actual German S3 guidelines, a spontaneous pneumothorax (SP) is considered as large when the sum of the interpleural distances derived from the Collins method is ≥4 cm [1]. Hence, in this study, we considered a spontaneous pneumothorax to be large at a size of ≥4 cm.

At the initial presentation of SSP, identified patients received chest tube (CT) treatment. Patients who were initially successfully treated with CT or were unsuitable for surgery (high Charlson Comorbidity Index score or poor ECOG status) and underwent only CT treatment during our study period were classified in the CT group. For patients suitable for surgery, indication for surgical therapy (VBPP) included persistent air leak for more than 5 days following CT treatment (*n* = 16) and ipsilateral or contralateral recurrent pneumothorax (*n* = 20). Prior to surgery, a computer tomography of the chest was performed to detect the cause of SSP and to determine the extent of a bullous disease. A team of three specialized thoracic surgeons (W.-T.K., A.S., A.R.) made an indication for surgery. Of note, indication for surgery was made individually depending on the comorbidity and underlying pulmonary disease as well as the patient’s choice. Patients with incomplete follow-up data and patients who received other treatment modalities (e.g., thoracotomy, apical pleurectomy, observation, needle aspiration) were excluded from this study. The primary endpoint of this study was to assess and compare the clinical outcomes and long-term recurrence rates after VBPP and CT treatment in our institution. Additionally, we analyzed underlying clinical parameters to determine potential risk factors for disease recurrence in our patient cohort. Recurrence was described as an ipsilateral or contralateral pneumothorax detected on a chest radiograph or computed tomography of the lung at presentation in our emergency room after treatment by VBPP or CT. The local ethics committee of the Heinrich-Heine University Clinic of Duesseldorf approved this study (study-no: 2020-1271, date of approval: 11.01.2021).

### 2.1. Surgical Technique: VATS-Bullectomy with Partial Pleurectomy (VBPP)

A team of three thoracic surgeons (A.S., A.R., and W.-T.K.) performed all surgical procedures and postoperative patient follow-up. All the patients were treated under general anesthesia with a double-lumen tube intubation and single-lung ventilation. After lateral positioning of the patient, video-assisted thoracoscopic surgery (VATS) was performed in the conventional two- or three-port approach. The initial thoracoscopy was undertaken for thorough inspection of the visceral and parietal pleura. The bullectomy was carried out by wedge resection using an endoscopic stapling device (Autosuture GIA Universal; COVIDIEN^TM^, Mansfield, MA, USA) when a ruptured bleb or bulla was identified. (For patients with extensive bullous disease, only the ruptured bleb/bulla and ultrathin bulla with a high risk of rupture were resected.) Only one patient underwent anatomical lung resection for stage I lung cancer. A partial pleurectomy was performed beginning from the apex of the pleural cavity. During this procedure, the parietal pleura was carefully separated from the endothoracic fascia while sparing the region of the subclavian artery and vein to avoid injury of these structures. A pleurectomy was performed up to the 7th or 8th intercostal space in a blunt manner. After cautious haemostasis of the endothoracic fascia using electrocautery to reduce the risk of hemothorax, one 24 Fr. chest tube was inserted and connected to a digital underwater seal system (Thopaz^+^, Medela AG, Baar, Switzerland) with a suction of −20 mm Hg. During postoperative care, the chest tube drain was removed when no clinical signs of air leak and a drain output of less than 200 mL after 24 h was evident. After the chest tube removal, a chest radiograph was taken to verify full expansion of the lung. All the patients received our standard postoperative medication regimen of analgesia (non-opioid, orally, or intravenously). The patients received either Metamizol-Natrium 1000 mg, Paracetamol 1000 mg, or Ibuprofen 600 mg four times per day. In cases of persistent pain using the standard pain medication regimen, we applied Piritramide (opioid) 7.5 mg intravenously every 4–6 h on patient request.

### 2.2. Outpatient Care and Follow-Up

One week after discharge, the patients visited our outpatient clinic for postoperative control and follow-up. These visits continued at 3-month intervals for one year. A chest radiograph was taken at each visit. Patients were advised to visit our emergency room at any time they had symptoms related to recurrent pneumothorax, such as dyspnoea or chest pain. Recurrent pneumothorax was identified clinically in each case with a chest radiograph and a computed tomography of the lung. For patients who recurred after CT or VBPP treatment, a VBPP or re-VATS was performed, respectively, depending on the patient’s clinical condition, underlying pulmonary disease, and the patient’s choice. For long-term follow-up, patients were contacted and assessed with a questionnaire. 

### 2.3. Statistical Analysis

Continuous data were tested for normal distribution using the Shapiro–Wilk test. Subsequently, the two-sample *t*-test was used for normally distributed data. For comparison of non-normally distributed data, the Mann–Whitney U test was applied. For categorical data, the chi-square test was used. Recurrence-free survival (RFS) was defined as the time between initial treatment by surgery (VBPP) or chest tube drainage (CT) and the ipsilateral or contralateral occurrence of recurrent pneumothorax. To identify potential risk factors for recurrent pneumothorax, Kaplan–Meier curves were generated and evaluated using the log-rank test. In addition, hazard ratios (HRs) with 95% confidence intervals (CIs) were estimated using a univariate Cox regression analysis. All variables potentially relevant to the development of recurrent secondary pneumothorax from a clinical point of view were then included into a multivariate Cox regression analysis. Statistical significance was assumed at *p* < 0.05. All data were analyzed using the SPSS 25.0 software program (Statistical Package for Social Sciences; SPSS Inc., Chicago, IL, USA).

## 3. Results

Between January 2008 and December 2020, 90 patients with secondary spontaneous pneumothorax (SSP) were treated either by VATS-bullectomy with partial pleurectomy (VBPP) or by chest tube (CT) in our institution. Eight patients were lost during follow-up and were excluded from this study. A total of 82 patients with a median age of 65 years (range 41–86) were included in this study. Thirty-six patients underwent VBPP, while 46 patients received CT treatment. The clinical characteristics of the patients and etiological causes of the SSP are summarized in Table 1.

Clinical variables, such as age, gender, BMI, pneumothorax size, COPD stage, ECOG status and Charlson Comorbidity Index, were similar in both groups (Table 1). However, two patients underwent VBPP, whereas 10 patients received CT treatment despite a Charlson Comorbidity Index score of five. VBPP was performed in this case on the patient’s choice despite the associated high risk of one-year mortality. Post-treatment complications such hemothorax (VBPP vs. CT: 13.9% vs. 2.8%, *p* = 0.43) and acute pneumonia (VBPP vs. CT: 30.6% vs. 10.9%, *p* = 0.026) were significantly more common in the VBPP cohort. Three patients who suffered a hemothorax in the VBPP group were successfully treated conservatively, while two patients underwent re-VATS. The patient who suffered a hemothorax after CT treatment was conservatively treated. In both groups, patients with acute pneumonia were treated successfully with our standard regimen of antibiotics. The high rate of acute pneumonia in the VBPP group seems to be related to postoperative pain, which might have impaired breathing exercises during the first postoperative days. During the clinical course, none of the patients died after treatment in both groups. Additionally, the mean length of hospital stay (LOS) was significantly longer in the VBPP group compared with the CT group (VBPP vs. CT: 9.3/14.1; *p* = 0.006). However, it should be considered that the LOS in the VBPP group was significantly prolonged by the preoperative period until surgery was performed (mean of 7.1 days) (Table 1). 

During a median follow-up period of 76.5 months (range 1–155 months), patients who underwent CT treatment experienced a significantly higher recurrence rate compared with patients following VBPP (VBPP vs. CT: 16.7% vs. 41.3%; *p* = 0.016). Interestingly, male gender was associated with a significantly higher rate of recurrence compared to female gender (male vs. female: 41.3% vs. 16.7%; *p* = 0.016) (Table 2).

**Figure 1:**
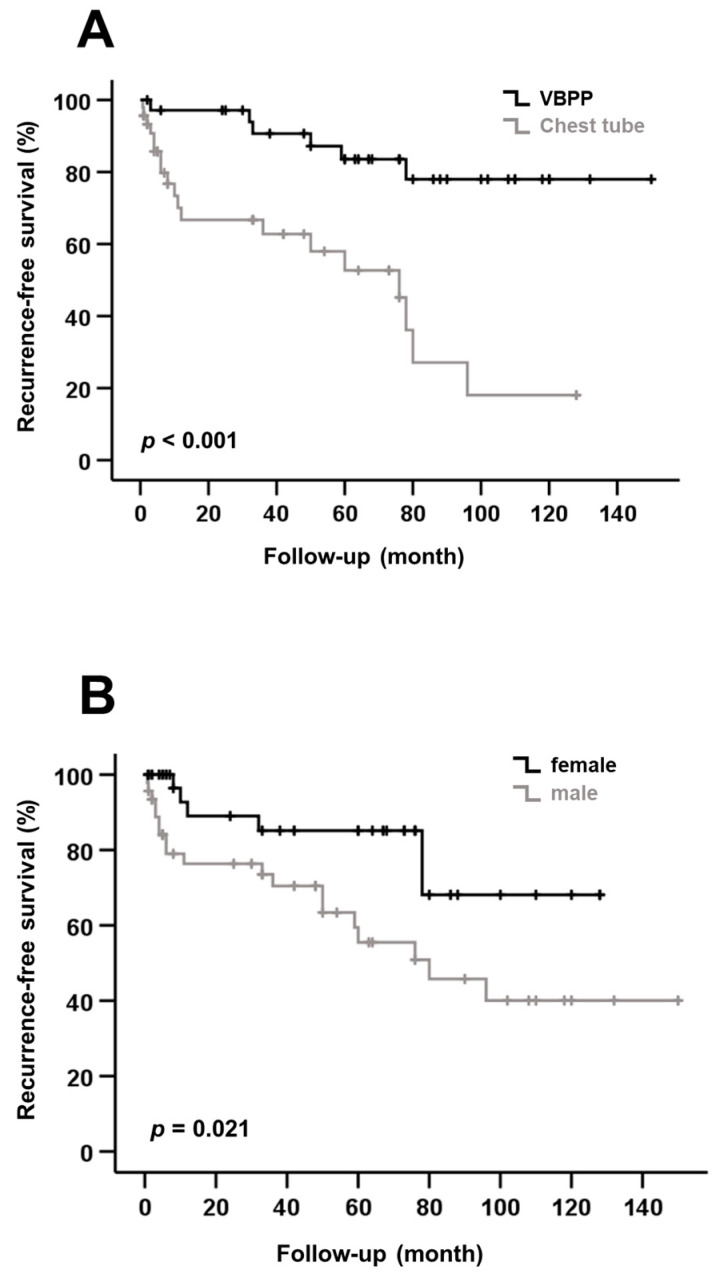
(**A**) Kaplan–Meier curve shows recurrence-free survival (RFS) after treatment by VATS-bullectomy with partial pleurectomy (VBPP) or chest tube (CT). VBPP was associated with significantly better RFS compared to CT treatment. (**B**) Male sex was associated with a significantly shorter RFS compared with female sex.

Moreover, we investigated the potential risk factors for the recurrence of secondary spontaneous pneumothorax (SSP) in our patient cohort. A univariate analysis revealed that the treatment of SSP with VBPP (CT vs. VBPP: HR 0.196; CI: 0.077–0.498; *p* < 0.001) and female sex (female vs. male: HR 2.803; CI: 1.118–7.030; *p* = 0.021) were associated with a significantly lower risk of SSP recurrence (Table 3; Figure 1A,B). Interestingly, the treatment modality chosen (CT vs. VBPP: HR 0.151; CI: 0.051–0.441; *p* = 0.001) was confirmed as the only independent predictive marker of SSP recurrence in the multivariate Cox regression analysis (Table 4).

## 4. Discussion

Due to the underlying pulmonary disease, secondary spontaneous pneumothorax (SSP) treatment requires a multimodal therapy concept. The challenging therapy aspect relies not only on the treatment of the pneumothorax, but also on the treatment of the underlying pulmonary disease. The actual guidelines recommend chest tube (CT) treatment for the initial SSP therapy. However, CT treatment has been reported to be associated with a high morbidity and recurrence rate [12,13]. Even after surgery, high morbidity and mortality rates have been reported [3,10,14]. This high morbidity and mortality rate depends not only on the underlying pulmonary disease, but also on the surgical technique implemented. Nowadays, VATS-bullectomy with partial pleurectomy (VBPP) is an increasingly used und well-established surgical technique for complicated spontaneous pneumothorax. In the treatment of primary spontaneous pneumothorax (PSP), VBPP has been well-reported to be effective and is associated with low rates of recurrence compared to VATS-apical pleurectomy, VATS-talc pleurodesis and bullectomy alone [15,16,17,18]. For treatment of SSP, reports on clinical outcomes and recurrence rates following VBPP are rare. Additionally, studies that evaluate potential risk factors for SSP recurrence are lacking in the literature. Therefore, the primary aim of this study was to evaluate and compare the long-term recurrence rates of patients treated either by VBPP or by CT in our institution. Moreover, we examined underlying clinical factors that might influence SSP recurrence in our patient cohort.

In our study, a total of 82 SSP patients were included. Chronic obstructive pulmonary disease (COPD) was the main cause of SSP (*n* = 64), followed by tuberculosis (*n* = 11) and lung cancer (*n* = 7). The high number of COPD patients in our study confirms the observations of the current guidelines that COPD is the main etiological cause of SSP [1,5,6]. Thirty-six patients with a median age of 65 years underwent VATS-bullectomy with partial pleurectomy (VBPP), while 46 patients (median age 60.5 years) were successfully treated by chest tube (CT) only. During a median follow-up period of 76.5 months (range 1–155 months), patients who underwent CT treatment experienced a significantly higher recurrence rate compared with patients following VBPP (VBPP vs. CT: 16.7% vs. 41.3%; *p* = 0.016). This difference was statistically significant in the univariate (CT vs. VBPP: HR 0.196; CI: 0.077–0.498; *p* < 0.001) and multivariate Cox regression analyses (CT vs. VBPP: B: −1.893; SE: 0.548; HR 0.151; CI: 0.051–0.441; *p* = 0.001). These results confirm that treatment by VBPP is associated with significantly lower SSP recurrence. The rate of SSP recurrence after VBPP in our study was significantly lower than the only comparable long-term study we found in the literature [17]. In this study of Shaikhrezai K et al. [17], five SSP patients underwent VBPP, of which two (40%) patients recurred during a median follow-up period of 73 months. Interestingly, our results of recurrence after VATS with mechanical pleurodesis (VBPP) is similar to the results of a previously reported study by Kim SJ et al. [19]. In this study, 22 patients after VATS-chemical pleurodesis (Talc–pleurodesis) displayed a significantly low rate of recurrence compared to 32 patients who underwent chemical pleurodesis via chest tube only. Our results and those of Kim SJ et al. confirm the efficacy of VATS with additional pleurodesis (chemical or mechanical) as a surgical technique to reduce recurrent disease and also demonstrate the ineffectiveness of chest tube drainage only as well as pleurodesis via chest tube drainage as treatment techniques to prevent recurrence. 

Compared with the CT group, patients in the VBPP group had a significantly longer length of hospital stay (LOS) (VBPP vs. CT: 14.1 days vs. 9.3 days, *p* = 0.006) and higher post-treatment complications (five patients suffered a hemothorax and two patients had an acute pneumonia; Table 1). The LOS in the VBPP group was significantly prolonged by the number of hospital days until surgery was performed (mean of 7.1 days). Additionally, a long recovery period after surgery due to the underlying pulmonary disease and the high rates of post-treatment complications certainly affected the LOS in the VBPP group. The LOS in both groups was comparable to previously reported studies in the literature [17,20]. With regard to complication rates, the number of patients who suffered a hemothorax was significantly higher in the VBPP group compared to patients after CT treatment (VBPP vs. CT: 13.9% vs. 2.8%, *p* = 0.043). We assume that the extent of pleurectomy during VBPP markedly influenced the occurrence of hemothorax. Two patients underwent re-VATS for hemothorax in the VBPP group, whereas three patients were successfully treated conservatively. We also observed a significantly higher rate of acute pneumonia in the VBPP group compared with the CT group (VBPP vs. CT: 30.6% vs. 10.9%, *p* = 0.026). This high rate of acute pneumonia in the VBPP group could be related to post-treatment pain, which might have impaired early breathing exercises and mobilization during the first postoperative days. In both groups, the patients were successfully treated with our standard regimen of antibiotics. During the clinical course, we observed no cases of in-hospital mortality in either group.

Analyses of potential risk factors for SSP recurrence, such as pneumothorax size, age, gender, BMI, COPD, smoking status, and treatment modality, revealed that male sex and treatment by chest tube (CT) were associated with a high risk of disease recurrence. As mentioned above, only treatment by CT was proven to be an independent risk factor for SSP recurrence in the multivariate Cox regression analysis.

The power of our study is limited due to its retrospective design, the lack of a surgical control group (e.g., VATS-apical pleurectomy, VATS-pleural abrasion) or a control group following chemical pleurodesis (e.g., VATS-Talc pleurodesis, chest tube-Talc/Doxycycline pleurodesis). Additionally, the small number of patients included limited possible subgroup analyses. Nevertheless, our results suggest that treating SSP patients with VATS-bullectomy and partial pleurectomy (VBPP) significantly reduces the long-term risk of disease recurrence compared with chest tube placement alone. It is associated with very low in-hospital mortality (in our study 0%). However, this advantage seems to be bought by a higher complication rate and a longer length of hospital stay. A prospective randomized trial with a large number of patients is needed to verify this observation

## 5. Conclusions

VBPP is a suitable surgical treatment for SSP. It is associated with a very low risk of disease recurrence. Therefore, VBPP should be offered as a treatment modality for patients with complicated SSP (recurrence or persistent air leak). However, prior to surgery, the underlying pulmonary disease, Charlson Comorbidity Index, and ECOG status should be considered, as these factors might prolong the length of hospital stay and negatively impact the patient as well as the surgical outcome.

## Figures and Tables

**Table 1:** Clinical characteristics and cause of SSP of the VBPP and CT groups.

Variables	VBPP (*n* = 36)	CT (*n* = 46)	*p*-Value
Gender			
Male	19 (52.8%)	27 (58.7%)	
Female	17 (47.2%)	19 (41.3%)	0.592
Smoking status			
Smokers (current and past)	30 (83.3%)	31 (67.4%)	
Non-smokers	6 (16.7%)	15 (32.6%)	0.101
Pneumothorax size (cm)			
Collins < 4 cm	5 (13.9%)	4 (8.7%)	
Collins ≥ 4 cm	30 (83.3%)	41 (89.1%)	
Missing	1 (2.8%)	1 (2.2%)	0.449
Age (yrs.)			
Median (Range)	65 (41–87)	60.5 (41–92)	0.144
Height (m)			
Mean (SD)	1.73 (0.08)	1.72 (0.08)	0.498
Weight (kg)			
Mean (SD)	65.8 (10.51)	64.7 (11.39)	0.655
BMI (kg/m^2^)			
Median (Mean)	21.5 (22)	20.8 (21.8)	0.483
ECOG status			
Grade 0–1	27 (75%)	23 (50%)	
Grade 2–3	8 (22.2%)	12 (26.1%)	
Grade 4	1 (2.8%)	11 (23.9%)	0.238
Charlson Comorbidity Index (score)			
1–2	20 (55.6%)	24 (52.2%)	
3–4	14 (38.9%)	12 (23.1%)	
5	2 (5.5%)	10 (24.7%)	0.302
Length of hospital stay (LOS) (days)			
Mean (SD)	14.1 (8.99)	9.3 (4.82)	0.006 *
Days until operation			
Mean (SD)	7.1 (2.65)	/	/
Complications			
Hemothorax	5 (13.9%)	1 (2.8%)	0.043 *
Acute pneumonia	11 (30.6%)	5 (10.9%)	0.026 *
Clavien-Dindo grade			
Grade I	0 (0%)	0 (0%)	
Grade II	14 (87.5%)	6 (100%)	
Grade IIIa	2 (12.5%)	0 (0%)	0.375
In-hospital mortality	0 (0%)	0 (0%)	1.00
COPD stage			
No COPD	7 (19.4%)	11(23.9%)	
COPD Gold I	5 (13.9%)	2 (4.3%)	
COPD Gold II	6 (16.7%)	3 (6.5%)	
COPD Gold III	13 (36.1%)	21 (45.7%)	
COPD Gold IV	5 (13.9%)	9 (19.6%)	0.282
**Cause of SSP**			**Total (N)**
COPD	29 (80.6%)	35 (76.1%)	64
Tuberculosis	6 (16.7%)	5 (10.9%)	11
Lung cancer	1 (2.7%)	6 (13%)	7

Data are presented as numbers and percent or median and mean. kg: kilogram, COPD: chronic obstructive pulmonary disease, m: metre, cm.: centimetre, yrs.: years, CT: chest tube, SSP: secondary spontaneous pneumothorax, VBPP: VATS-bullectomy with partial pleurectomy, N = sum of the number of patients in both groups. * *p*-value < 0.05 indicates statistical significance.

**Table 2:** Patient clinical characteristics and recurrence rates of SSP.

Variable	Recurrence *n* (%)	*p*-Value
Gender		
Male	19 (41.3)	
Female	6 (16.7)	0.016 *
Age		
≤median (63 years)	14 (31.8)	
>median (63 years)	11 (28.9)	0.778
BMI		
≤median (21.1 kg/m^2^)	13 (31.0)	
>median (21.1 kg/m^2^)	12 (30.0)	0.925
Smoking status		
Smokers (past and current)	8 (38.1)	
Non-smokers	17 (27.3)	0.380
Treatment		
VBPP	6 (16.7)	
CT	19 (41.3)	0.016 *
Pneumothorax size		
Collins < 4 cm	3 (33.3)	
Collins ≥ 4 cm	20 (28.2)	0.747
Side of recurrence at presentation		
Ipsilateral	12 (60)	
Contralateral	8 (40)	0.211
COPD stage		
No COPD	4 (22.2)	
COPD Gold I-IV	21 (32.8)	0.389
Other causes of SSP		
Tuberculosis	4 (22.2)	
Lung cancer	0 (77.8)	0.024 *

Data are presented as numbers and percent, median and range (for non-normally distributed data) and mean and standard deviation (SD) (for normally distributed data). kg: kilogram, COPD: chronic obstructive pulmonary disease, m: metre, cm.: centimetre, yrs.: years, CT: chest tube, VBPP: VATS-bullectomy with partial pleurectomy, N = sum of the number of patients in both groups. * *p*-value < 0.05 indicates statistical significance.

**Table 3:** Univariate analysis of potential risk factors for recurrence of SSP.

Risk Factor	Hazard Ratio	95% CI	*p*-Value
Gender			
Female vs. Male	2.803	1.118–7.030	0.021 *
Age			
≤63 years vs. >63 years	0.811	0.368–1.790	0.602
BMI			
≤21.1 kg/m^2^ vs. >21.1 kg/m^2^	1.073	0.489–2.355	0.861
Smoking status			
Non-Smokers vs. Smokers	0.690	0.297–1.601	0.382
Treatment			
CT vs. VBPP	0.196	0.077–0.498	<0.001 *
Pneumothorax size (cm)			
Collins < 4 vs. Collins ≥4	1.075	0.318–3.630	0.907
COPD stage			
COPD vs. no COPD	1.051	0.358–3.082	0.927

Univariate analysis displays potential risk factors for the recurrence of SSP. Patients treated by chest tube (CT) and male patients had a significantly higher risk of SSP recurrence. * *p*-value < 0.05 indicates statistical significance. VBPP: VATS-bullectomy with partial pleurectomy, COPD: chronic obstructive pulmonary disease, BMI: body mass index, CI: confidential interval, SSP: secondary spontaneous pneumothorax.

**Table 4:** Multivariate Cox regression analysis of potential risk factors for recurrence of SSP.

Risk Factor	B	SE	Wald	Hazard Ratio	95% CI	*p*-Value
Gender						
Female vs. Male	0.931	0.494	3.544	2.536	0.962–6.684	0.060
Age						
≤63 years vs. >63 years	0.005	0.018	0.065	1.005	0.969–1.041	0.799
BMI						
≤21.1 kg/m^2^ vs. >21.1 kg/m^2^	0.237	0.487	0.237	1.268	0.488–3.294	0.627
Smoking status						
Non-smokers vs. Smokers	−0.090	0.500	0.032	0.914	0.343–2.437	0.857
Treatment						
CT vs. VBPP	−1.893	0.548	11.938	0.151	0.051–0.441	0.001 *
Pneumothorax size (cm)						
Collins < 4 vs. Collins ≥ 4	−0.433	0.738	0.345	0.648	0.153–2.755	0.557
COPD						
no COPD vs. COPD	0.610	0.685	0.793	1.840	0.481–7.041	0.373

Multivariate Cox regression analysis displayed the treatment modality chosen (CT or VBPP) as the only independent risk factor for SSP recurrence. VATS-bullectomy with partial pleurectomy (VBPP) treatment was associated with a significantly lower risk of SSP recurrence. * *p*-value < 0.05 indicates statistical significance. VBPP: VATS-bullectomy with partial pleurectomy, CI: confidential interval, BMI: body mass index, B: coefficient beta, SE: standard error, Wald: Wald’s statistics.

## Data Availability

The data presented are included in this study; the corresponding author on request may provide additional data.
